# Specific plasma microRNA profiles could be potential non-invasive biomarkers for biochemical pregnancy loss following embryo transfer

**DOI:** 10.1186/s12884-024-06488-x

**Published:** 2024-05-08

**Authors:** Lang Shen, Hong Zeng, Yu Fu, Wenmin Ma, Xiaoling Guo, Guoqun Luo, Rui Hua, Xiaocong Wang, Xiao Shi, Biao Wu, Chen Luo, Song Quan

**Affiliations:** 1 Reproductive Center of Gynecology and Obstetrics Department, NanFang Hospital, Southern Medical University, Guangzhou, 510515 China; 2 Department of Reproductive Medicine Center, Foshan Maternal and Child Health Care Hospital, Southern Medical University, Foshan, 528000 China

**Keywords:** Biochemical pregnancy loss, Micro RNA, Non-invasive biomarker, Embryo implantation, Assisted reproductive technology

## Abstract

**Background:**

Plasma microRNAs act as biomarkers for predicting and diagnosing diseases. Reliable non-invasive biomarkers for biochemical pregnancy loss have not been established. We aim to analyze the dynamic microRNA profiles during the peri-implantation period and investigate if plasma microRNAs could be non-invasive biomarkers predicting BPL.

**Methods:**

In this study, we collected plasma samples from patients undergoing embryo transfer (ET) on ET day (ET0), 11 days after ET (ET11), and 14 days after ET (ET14). Patients were divided into the NP (negative pregnancy), BPL (biochemical pregnancy loss), and CP (clinical pregnancy) groups according to serum hCG levels at day11~14 and ultrasound at day28~35 following ET. MicroRNA profiles at different time-points were detected by miRNA-sequencing. We analyzed plasma microRNA signatures for BPL at the peri-implantation stage, we characterized the dynamic microRNA changes during the implantation period, constructed a microRNA co-expression network, and established predictive models for BPL. Finally, the sequencing results were confirmed by Taqman RT-qPCR.

**Results:**

BPL patients have distinct plasma microRNA profiles compared to CP patients at multiple time-points during the peri-implantation period. Machine learning models revealed that plasma microRNAs could predict BPL. RT-qPCR confirmed that miR-181a-2-3p, miR-9-5p, miR-150-3p, miR-150-5p, and miR-98-5p, miR-363-3p were significantly differentially expressed between patients with different reproductive outcomes.

**Conclusion:**

Our study highlights the non-invasive value of plasma microRNAs in predicting BPL.

**Supplementary Information:**

The online version contains supplementary material available at 10.1186/s12884-024-06488-x.

## Background

Embryo implantation is the speed-limit step of in-vitro fertilization and embryo transfer (IVF-ET). It is reported that the successful implantation rate is approximately 30%. About 70% of embryos result in pregnancy loss before live birth. Pregnancy loss occurs in four stages: implantation failure, biochemical pregnancy loss (BPL), early miscarriage, and late miscarriage. Implantation failure occurs before the embryo starts implantation with a negative hCG test, which accounts for 30% of embryos' fate. BPL and early miscarriage together constitute early pregnancy loss. Late miscarriage occurs after 12 gestational weeks. Unlike early miscarriage, most studies conclude that the leading causes of BPL are endometrial factors rather than embryonic factors [1–6]. On the contrary, more than 50% causes of early miscarriage are abnormal embryo karyotypes. Although the role of embryological factors in BPL cannot be ruled out, at least embryological factors may not be the dominant factor. Besides, immune factors may play an essential role in developing BPL, as immunotherapy dramatically reduces the BPL rate [7].

Until now, BPL has no conclusive diagnostic criteria, such as hCG threshold and detection time-point [8]. The terminologies used to describe BPL are inconsistent. BPL is also named as biochemical pregnancy [3, 9], early pregnancy loss [10], occult pregnancy [11], preclinical pregnancy loss/abortion [2], premenstrual pregnancy loss [12], and non-visualized pregnancy loss [13]. In summary, BPL refers to a situation in which the pregnancy losses after one or more positive hCG tests followed by decreased hCG levels, which does not result in the visualization of an intrauterine or ectopic gestational sac. In contrast to implantation failure, BPL embryos start implantation; unlike early miscarriage, BPL experiences a very early pregnancy loss during implantation, which is hard to notice in a natural pregnancy. Assisted reproduction technology (ART) has led to the discovery of more BPLs, as patients are closely monitored after ET. The BPL rate is reported to be ~10%. The exact etiology of BPL is poorly understood because of the unavailability of embryonic tissues. With the increasing application of high-throughput sequencing technology in medical diagnosis, it has been found that BPL may have a specific endometrial expression profile compared to negative pregnancy or clinical pregnancy [9]. Díaz-Gimeno et al. reported that 95 genes in the endometrium could be potential indicators of BPL [9]. Identifying particular expression profiles and biomarkers by high-throughput techniques may provide a pathological basis, prevention, and treatment of BPL. However, endometrial biopsy is invasive and cannot be performed in the ET cycle. Besides, the same patient may have different transcriptomic profiles in different menstrual cycles due to psychological, environmental, and therapeutic effects. Transcriptomic profiles in the non-ET cycle may bring biased guidance in the subsequent ET cycle. Therefore, exploring non-invasive biomarkers of BPL in the ET cycle is needed. However, choosing what samples and omics for detection are challenging. Firstly, we choose the peripheral plasma as the sample for high-throughput omics detection because peripheral blood is easy to obtain in the ET cycle before or after ET compared to traditional endometrial biopsy or uterine fluid aspiration. Secondly, we choose miRNA-sequencing to detect miRNA profiles in the peripheral plasma because miRNAs are stable and are highly valuable in predicting or diagnosing particular diseases. Moreover, compared to serum, the cell-free plasma may provide the most reliable-to-interpret data from circulating miRNAs [14].

MicroRNAs (miRNAs) are small, non-coding functional RNAs that are ~22 nucleotides in length, which regulate the expression of target mRNA by binding the 3′-untranslated region (3′-UTR) [15]. Unlike mRNAs, miRNAs are highly stable and can be detected in various fluids, including semen, blood, saliva, uterine fluid, amniotic fluid, and breast milk. It is well-studied that circulating miRNAs can act as non-invasive biomarkers in various cancers. A miRNA expression signature in the endometrial tissues predictive of recurrent implantation failure has been described [16]. Some studies indicate that miRNAs in the culture medium or peripheral plasma have the potential as non-invasive biomarkers for embryo implantation capacity [17–20].

Before embryo implantation, the endometrium undergoes a morphological, functional, and molecular transition to allow endometrium for embryo implantation. Abnormality at this stage may lead to pregnancy loss. The plasma's miRNA profiles at the implantation window may partially reflect the state of the endometrium as endometrium can secrete miRNAs into the plasma. Except for endometrium-enriched miRNAs in plasma, some miRNAs derived from immune cells, blood cells, or even other tissues are related to certain pathophysiological, psychological, iatrogenic, or environmental conditions. Those conditions may also affect the uterine environment for embryo implantation. Therefore, the plasma miRNA profile reflects the comprehensive situation under various effects. Whether the miRNA profiles at the ET day have the potential to predict BPL needs to be studied in the current study. Moreover, the embryo also secretes miRNAs at an early stage of implantation, which can be detected in the plasma. The dynamic miRNA profiles at different time-points during the peri-implantation phase may reflect the endometrium-embryo interactions. A recent study described the miRNA profiles in maternal plasma from early to mid-gestation [21]. However, dynamic plasma miRNA profiles during the peri-implantation period have not been investigated. It is reported that miRNAs in the culture medium could be potential biomarkers for clinical outcomes of ART [22]. However, miRNAs secreted by embryos only reflect the embryo factors that account for implantation failure. The endometrium factor or other systematic factors are not included. In this study, we detected the miRNA profiles at different time-points (ET day, 11 days after ET, and 14 days after ET) and analyzed the dynamic plasma miRNA signatures during the peri-implantation period. We established a predictive model for BPL with machine learning technology and validated the sequencing results with Taqman RT-qPCR.

## Methods

### Patients, samples, definitions, and ethics

In the miRNA-sequencing, we recruited patients who underwent routine IVF-ET at the Reproductive Medicine Center of Nanfang Hospital from October 2020 to June 2021. The inclusion criteria were: regular menstrual cycle, transferred 1~2 good-quality embryos. The exclusion criteria were: PGD cycles, oocyte donor cycles, combined with endometriosis or PCOS, uterine abnormality, endocrine diseases, or abnormal karyotypes. Peripheral plasma samples were collected on the day of embryo transfer (ET) before ET (ET0), 11 days after ET (ET11), and 14 days after ET (ET14). The patients were followed up for reproductive outcomes and divided into the negative pregnancy group (NP), biochemical pregnancy loss group (BPL), or clinical pregnancy group (CP) according to serum hCG levels at ET11~14 combined with ultrasonography. The shortest follow-up is at least 28 days after ET to confirm clinical pregnancy. No patient lost follow-up at this stage. As the incidence rates of BPL, NP, and CP are very different, a total of ten patients developed BPL in the study period we matched with NP and CP patients at a ratio of 1:1:1. The 30 case-matched samples were sent for miRNA-sequencing. Blood samples were collected at three time-points (ET0, ET11, and ET14) from BPL patients and CP patients, samples were collected at two time-points (ET0 and ET11) from NP patients. Two patients lack samples at ET 14. Finally, a total of 78 plasma samples were sent for miRNA sequencing. In the RT-qPCR validation, all 18 BPL patients during the study period were included. The BPL patients were matched with NP and CP patients at a ratio of 1:1:2 by propensity score matching (PSM) to control confounding factors that may affect pregnancy outcomes. The variables included in the PSM are female age, BMI, endometrial thickness, cycle protocol, number of transferred embryos, and embryo stage. Patients who developed early miscarriages were excluded from the RT-qPCR validation analysis. All patients included in this study are under the same hormonal treatment in the luteal phase support.

Morphological good-quality embryos are defined as the following: for Day 3 cleavage stage embryos, the embryos were graded according to the number and shape of blastomeres, cytoplasmic granules, and cytoplasmic fragments based on the Istanbul consensus [23]. Grade I and Grade II embryos with 7-9 blastomeres on Day 3 were defined as good-quality embryos; for Day 4 compact/morula stage embryos, the embryos were graded according to compaction, fragmentation, and vacuoles by a modified SART system [24, 25], the compacting C1 or compacted C2 or morula without vacuoles, and fragmentation<25% were defined as good-quality embryos. For Day 5 or Day 6 blastocyst stage embryos, the blastocysts were graded according to Gardner's system [26]. Blastocysts ≥ 3AA, 3AB, 3BA, and 3BB are defined as good-quality embryos. NP was defined as hCG <10 IU/L at ET11. BPL was defined as the pregnancy losses after a positive hCG test at ET11 without visualizing the gestational sac or any sign of ectopic pregnancy. CP was confirmed by the ultrasonographic gestational sac.

Peripheral blood samples (~10ml per patient) were obtained from the peripheral vein into the Ethylene Diamine Tetraacetic Acid (EDTA) tubes. The tubes were inverted five times, stored on ice, and processed within 30 min. Each sample was centrifuged at 1500g for 15 min at 4°C to separate plasma from cells. The supernatant was then collected from each tube and transferred to new tubes. The plasma was stored at -80 °C until further miRNA sequencing or RT-qPCR validation. Each sample was freeze-thaw once.

The study was approved by the Ethics Committee of Nanfang Hospital (accession number NFEC-2021-135-1) and is in compliance with the principles of the Declaration of Helsinki. Informed consent was obtained from every participant.

### RNA isolation

The miRNAs were extracted from the plasma using the mirVana™ miRNA Isolation Kit (Cat #. AM1561, Austin TX, USA) according to the manufacturer’s protocol. The concentration and quality of RNA samples were determined by NanoDrop ND-1000 spectrophotometer (Thermo Fisher, Wilmington, DE, USA) and 2100 Bioanalyzer Instruments (Agilent Technologies, Santa Clara, CA, USA).

### MicroRNA sequencing

The microRNA-sequencing process was described before [20]. Briefly, the miRNA sequencing library was constructed using the QIAseq miRNA Library Kit (QIAGEN, German). The total RNA of each sample was used to prepare the miRNA sequencing library in the following steps: 3'-adaptor ligation, 5'-adaptor ligation, cDNA synthesis, PCR amplification, and gel purification. After quantification with Qubit (Thermo Fisher, USA), the libraries were captured on cBOT (Illumina, USA) to be amplified in situ as clusters. MiRNA-seq was performed with the Illumina NovaSeq 6000 (Illumina, USA). After sequencing, the adaptor sequences were trimmed, and the quality-filtered reads were harvested as clean reads using fastx (version 0.0.13). The clean reads were mapped to databases of the human genome, RFam, RepBase, mRNA database, and miRBase using the bowtie software, allowing up to one mismatch. Based on the miRNA biogenesis model, we used the miRCat software to predict novel miRNAs. The clean reads of each sample were aligned to merged miRNA databases (known miRNAs from miRBase plus the newly predicted miRNAs) to calculate the miRNA expression levels. The numbers of mapped tags were defined as the raw expression levels of the miRNAs.

### MicroRNA transcriptomic analysis

PCA analysis was performed with the TPM normalized and scaled data. The DESeq2 (version1.34.0) Package [27] was used to screen the differentially expressed miRNAs (DEmiRs) between the patients with different reproductive outcomes. The DEmiRs were selected by adjusted *p*-value < 0.05 and |fold change (FC)| >2. The target genes of miRNAs were predicted by miRTarBase (https://mirtarbase.cuhk.edu.cn/~miRTarBase/miRTarBase_2022/php/index.php). The GO and KEGG enrichment analysis of the target genes were performed using the clusterProfiler Package (version4.2.1).

### Time-course differential miRNA expression analysis

The time-course differential miRNA expression analyses were conducted between the BPL and CP groups at three time-points. Analyses were performed on the TPM normalized data. The maSigPro (version 1.66.0) Package [28] was used to perform the time-course analysis. The maSigPro modeling gene expression by polynomial regression and identifies expression changes along one or across several time series by introducing dummy variables in the model. The method progresses in two regression steps: the first one selects genes with non-flat profiles, and the second step creates the best regression models for each gene to identify the specific time-associated changes. The cut-off value for the R^2^ parameter in the second regression step is 0.2.

### Weighted gene co-expression network analysis

The co-expression network of the miRNAs was constructed by the WGCNA (version 1.71) Package [29] as previously described [20]. Briefly, WGCNA was performed on TPM-normalized data. MiRNAs expressed in less than five samples and less than five TPM reads were filtered, resulting in a dataset containing 564 miRNAs. The average-linkage hierarchical clustering method based on a minimum size (gene group) of 10 was employed to cluster all modules. The modules with high similarity were merged to obtain the co-expression network. Each module's miRNA with the highest connectivity was identified as the hub miRNA. The Cytoscape software (version 3.8.2) was used to visualize the miRNA co-expression network. Connectivity>0.3 was selected for visualization. Only the top ten miRNAs were visualized if there were more than ten miRNAs in the module.

### Predictive model for biochemical pregnancy loss

Twenty patients (ten BPL patients and ten CP patients) were included in the predictive model construction. The miRNA-seq datasets on the ET day were used to construct the predictive model. The 20 patients were split into the training set and the testing set. Fourteen patients included in the training set were used to establish the predictive model, and six patients included in the testing set were used to check the model performances. We used two methods to train the model. One is the Elastic Net Regression. The other one is the Random Forest method. As the sample size is small, we applied repeated cross-validation in the training process. We trained the model with seven-fold validation and repeated for three times. The glmnet (version 4.1-3) Package [30] was used to perform the Elastic Network Regression. The randomForest (version 4.7-1) Package [31] was used to perform the random forest modeling. The caret (version 6.0-92) Package [32] was used to select the best tuning parameters and run the repeated cross-validation.

### RT-qPCR validation

The reverse transcript was performed using the Taqman MicroRNA Reverse Transcription Kit (ABI, 4366597, USA). Stem-loop primer method was used for cDNA synthesis. Quantitative PCR was performed using the Taqman Universal PCR Master Mix (ABI, 4440049, USA) according to the manufacturer’s instructions and analyzed using the QuantStudio 5 Real-Time PCR System (ABI, USA). All miRNA assay primers used in this study were purchased commercially (Thermo Fisher Scientific, USA). The C. elegans-miR-39 mimic (QIAGEN, Hilden, Germany) was used as an endogenous control to normalize the relative expression levels of the miRNAs. Every sample was performed in triplicate, and the mean was used to determine the miRNA levels. The amplification efficiency for each miRNA was determined by equation (E=10^[-1/slope]^-1). Data were processed using the 2^(-ΔΔCt)^ method if the amplification efficiency of both target and reference genes was close to 100% (range from 90% to 110%) and the relative deviation was less than 5%, or else, Pfaffl method was applied [33]. Missing values, which resulted when no Ct value could be determined within 45 cycles, were excluded.

### Statistical analysis

We presented the normally distributed continuous variables as mean ± standard deviation (SD). Non-normally continuous variables were presented as median and interquartile ranges. Normally distributed data were compared using the t-test or ANOVA analysis, and non-normally distributed data using the Mann-Whitney U test or Wilcoxon test. A p-value less than 0.05 was considered to be statistically significant. All analyses were performed using the R software (version 4.1.3).

## Results

### BPL indicative microRNAs in plasma during the peri-implantation period

Ten patients in each group were included for miRNA-sequencing. The characteristics of the three groups are summarized in Table 1 (left 2~5 Columns). Age, BMI, endometrial thickness, number of transferred embryos, and embryo stage are not significantly different between the three groups.

**Table 1:** Characteristics of study population involved in miRNA sequencing and RT-qPCR validation

	**Patients involved in miRNA sequencing**				**Patients involved in RT-qPCR validation**			
	**NP**	**BPL**	**CP**	**p.overall**	**NP**	**BPL**	**CP**	**p.overal**
	*N*=10	*N*=10	*N*=10		*N*=18	*N*=18	*N*=32	
Age (year)	34.3 (6.95)	32.0 (4.62)	32.5 (3.21)	0.583	33.94 (5.27)	34.50 (5.12)	33.62 (3.57)	0.805
BMI (kg/m^2^)	22.3 (2.55)	21.2 (2.31)	21.9 (4.66)	0.748	21.69 (1.74)	21.38 (1.87)	21.45 (3.32)	0.934
Endometrial thickness (mm)	10.2 (3.22)	9.70 (1.95)	10.2 (3.19)	0.903	9.06 (2.04)	9.59 (2.24)	10.52 (2.26)	0.071
Number of ET				0.348				0.292
1	5 (50.0%)	5 (50.0%)	8 (80.0%)		5 (27.78%)	7 (38.89%)	6 (18.75%)	
2	5 (50.0%)	5 (50.0%)	2 (20.0%)		13 (72.22%)	11 (61.11%)	26 (81.25%)	
Stage of ET				0.404				0.081
Cleavage	0 (0.00%)	2 (20.0%)	3 (30.0%)		10 (55.56%)	3 (16.67%)	8 (25.00%)	
Morula	7 (70.0%)	4 (40.0%)	4 (40.0%)		6 (33.33%)	8 (44.44%)	17 (53.12%)	
Blastocyst	3 (30.0%)	4 (40.0%)	3 (30.0%)		2 (11.11%)	7 (38.89%)	7 (21.88%)	

*NP *Negative pregnancy, *BPL *Biochemical pregnancy loss, *CP *Clinical pregnancy, *ET *Embryo transfer

**Fig. 1:**
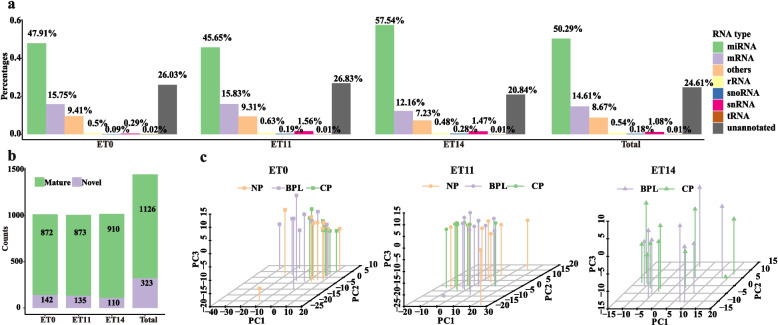
MicroRNA profiles in peripheral plasma during the peri-implantation period at different time-points. **a** The number of known miRNAs and novel miRNAs. **b** The proportion of small RNA types. **c** PCA plots

There are 1449 miRNAs detected in the 78 plasma samples (1126 known miRNAs and 323 novel miRNAs). There are 1014 miRNAs (872 known miRNAs and 142 novel miRNAs), 1008 miRNAs (873 known miRNAs and 135 novel miRNAs), and 1020 miRNAs (910 known miRNAs and 110 novel miRNAs) detected at ET0, ET11, and ET14, respectively (Fig. 1a). MiRNA accounts for 50.29% of all the small RNAs detected in all samples. MiRNA accounts for 47.91%, 45.65%, and 57.54% of all the small RNAs detected in the samples at ET0, ET11, and ET14, respectively (Fig. 1b). From ET0 to ET14, the total miRNA percentage and known miRNA percentage are increasing. PCA plot at ET0 showed that the BPL cluster is distinct from the CP cluster; however, the NP cluster is scattered. PCA plot at ET11 showed a similar tendency as ET0. However, at ET14, the PCA plot showed that the BPL cluster is not significantly distinct from the CP cluster (Fig. 1c).

**Fig. 2:**
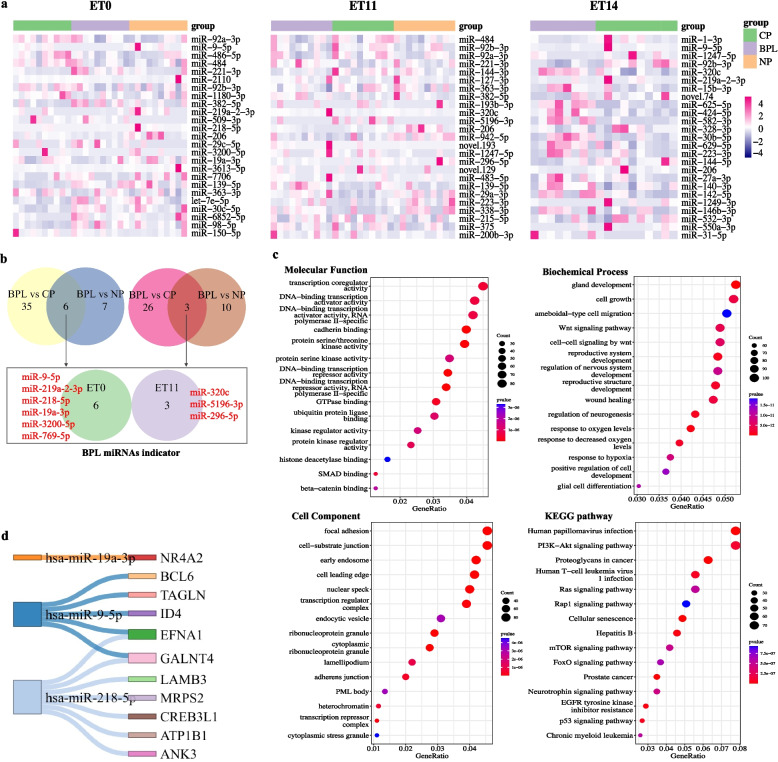
Comparison analysis of differentially expressed miRNAs (DEmiRs) in plasma with different reproductive outcomes at different time-points. **a** Heatmaps showing the DEmiRs between different reproductive outcomes at different time-points. **b** Venn plot showing the intersection of different DEmiRs comparisons at different time-points. **c** Gene ontology (GO) and KEGG pathway enrichment analysis of BPL-associated DEmiRs. **d** Interaction of BPL-associated DEmiRs with BPL-associated mRNAs. The DEmiRs lists of each comparison can be found in Supplementary Table 1

To describe the BPL plasma miRNA signature during the peri-implantation period, we analyzed differentially expressed miRNAs (DEmiR) by comparing BPL with CP (BPL versus CP) and BPL with NP (BPL versus NP) at ET0, ET11, and ET14, respectively. Heatmap (Fig. 2a) showed the top 25 DEmiRs between the BPL, CP, and NP groups at ET0, ET11, and ET14, respectively. At ET0, comparison with the CP profile identified 41 DEmiRs, and comparison with NP identified 13 DEmiRs; the intersection of the two comparisons results in 6 common DEmiRs (miR-9-5p, miR-219a-2-3p, miR-218-5p, miR-19a-3p, miR-3200-5p, miR-769-5p) (Fig. 2b). At ET11, comparison with the CP profile identified 29 DEmiRs, and comparison with NP identified 13 DEmiRs; the intersection of the two comparisons results in 3 common DEmiRs (miR-320c, miR-5196-3p, miR-296-5p) (Fig. 2b). At ET14, a comparison with the CP profile identified 28 DEmiRs. A total of 9 common DEmiRs in all comparisons (BPL vs CP, BPL vs NP) at ET0 and ET11 were defined as BPL-indicative miRNAs (Fig. 2b). Figure 2c shows the top 15 enriched molecular functions, biochemical processes, cell components, and KEGG pathways of the target genes of BPL-indicative miRNAs, the target genes of BPL-indicative miRNAs are enriched in processes related to gland development, reproductive system development, cell growth, migration, and adhesion. The enriched pathways include Wnt, PI3K-Akt, Ras, Rap1, mTOR, FoxO, and P53 signaling are closely related to embryo implantation. BPL-indicative mRNAs are extracted from the publication by Gimeno [9]. BPL-indicative mRNAs such as NR4A2, BCL6, TAGLN, ID4, EFNA1, GALNT4, LAMB3, MRPS2, CREB3L1, ATP1B1, ANK3 were targets of the BPL-indicative miR-19a-3p, miR-9-5p, and miR-218-5p (Fig. 2d). Results of all the DemiR comparisons at different time-points are listed in Supplementary Table 1.

### Time-dependent miRNA markers during the peri-implantation period

**Fig. 3:**
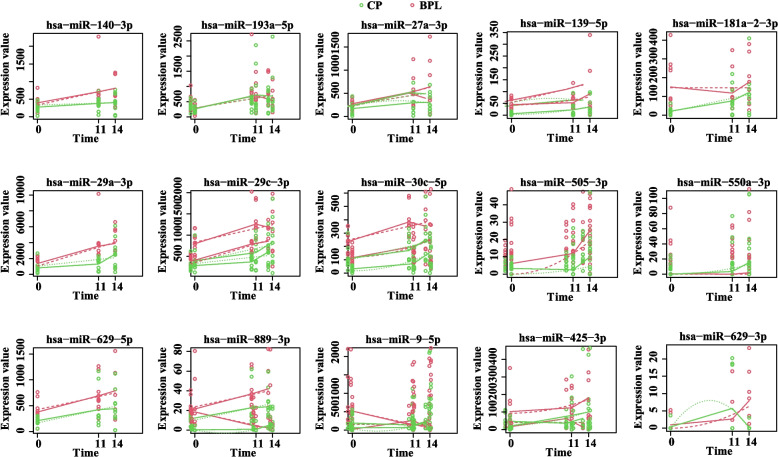
Time-course differential miRNA expression analysis by maSigPro. The green dots and lines represent the clinical pregnancy group, red dots and lines represent the biochemical pregnancy group. Fitted curves of the CP and BPL groups are displayed as green and red dotted lines, respectively

To identify the time-dependent miRNAs during the peri-implantation period, we performed the time-course analysis with miR-seq data of CP and BPL patients at three time-points following ET. MaSigPro analysis identified nine significant time-dependent miRNAs (miR-140-3p, miR-193a-5p, miR-27a-3p, miR-29a-3p, miR-29c-3p, miR-30c-5p, miR-629-5p, miR-889-3p, miR-9-5p) during the peri-implantation period, and 7 differential time-dependent miRNAs (miR-139-5p, miR-181a-2-3p, miR-425-3p, miR-505-3p, miR-550a-3p, miR-629-3p, miR-9-5p) were identified between the CP and BPL groups. In summary, most time-dependent miRNAs exhibited an increasing tendency from ET0 to ET14. However, the expression of the miRNAs at one or two points was differentially expressed. Some miRNAs exhibited different time-dependent tendencies between the BPL and CP groups; miR-889-3p in the BPL group exhibited marked downregulation from ET0 to ET14, while miR-889-3p in the CP group showed an increasing tendency; miR-9-5p in the BPL group exhibited an increasing tendency from ET0 to ET11, while miR-9-5p in the CP group exhibited a decreasing tendency; miR-181a-2-3p in the BPL group exhibited a decreasing tendency from ET0 to ET11, while miR-181a-2-3p in the CP group exhibited an increasing tendency. The maSigPro results are shown in Fig. 3.

### MiRNA co-expression network during the peri-implantation period

**Fig. 4:**
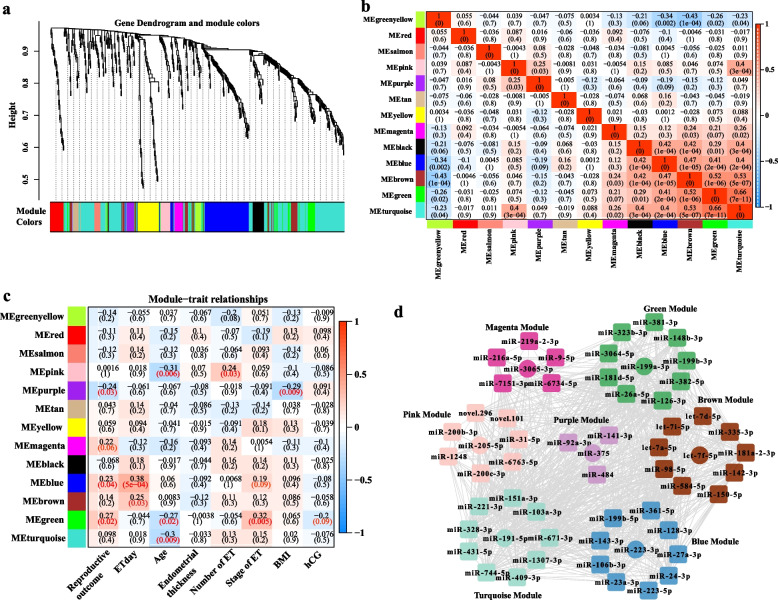
Weighed gene co-expression network analysis. **a **Clustering dendrograms of all miRNAs, with dissimilarity based on the topological overlap, together with assigned module colours. **b** Module correlations. **c** Module-trait associations. **d** miRNA co-expression network of different modules. The miRNA lists and hub miRNAs for each module can be found in Supplementary Table 2

564 miRNAs in 78 samples were subjected to WGCNA after removing miRNAs with low expression. We obtained 13 modules in total (Fig. 4a). The module-module relationship heatmap showed that the black, blue, brown, green, turquoise, and pink modules are closely correlated (Fig. 4b). The module-trait relationship heatmap showed the correlations of the models with reproductive outcomes and clinical traits (Fig. 4c). To understand the co-expression pattern of miRNAs in each meaningful module with the clinical trait, we select the meaningful module with the *p*-value<0.1 as the threshold. The green module, blue module, purple, and magenta modules are relevant to the reproductive outcomes; the blue and brown modules are relevant to ET day; the pink, green and turquoise modules are relevant to age; the purple module is relevant to BMI, the green module is relevant to hCG levels. The miRNA co-expression networks in each module are shown in Fig. 4d. The miRNA lists and hub miRNAs in each module can be found in Supplementary Table 2.

### Predictive models for BPL based on miRNA profile

**Fig. 5:**
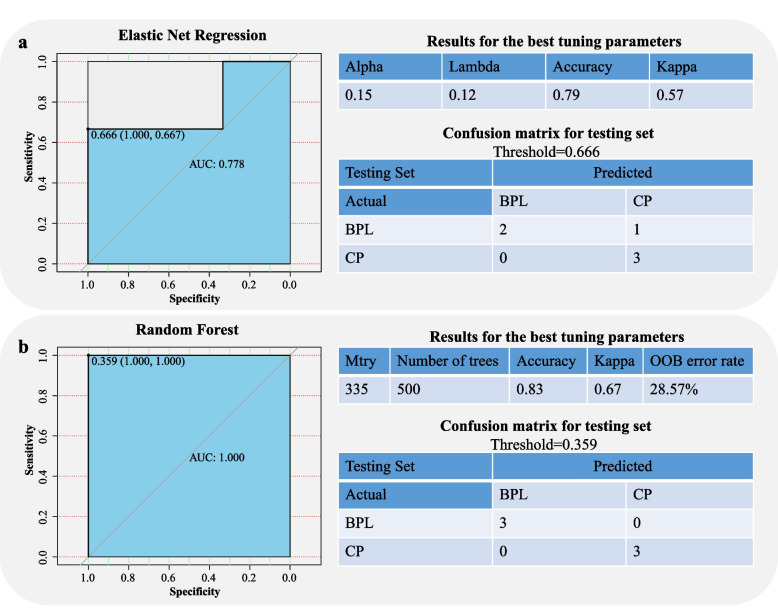
Performances of two predictive models for biochemical pregnancy loss. **a** ROC curve and confusion matrix of the Elastic Network Model. **b** ROC curve and confusion matrix of the Random Forest Model. The miRNA lists included in each model can be found in Supplementary Table 3

We construct the predictive model for BPL based on miRNA profiles on ET day using two training models. By the Elastic Net regression model, the best tuning parameters to train the model were alpha=0.15, lambda=0.12; results showed that the accuracy is 0.79 and the kappa is 0.57. All the 14 patients in the training set were accurately classified with the predictive model trained by Elastic net regression; in the testing set, the area under curve (AUC) is 0.778, the accuracy rate is 83.33%, sensitivity is 66.7%, specificity is 100% (Fig. 5a). For the Random Forest training model, the best tuning parameters chosen to train the model were mtry=335 and number of trees =500; results showed that the accuracy rate is 0.83, the kappa is 0.67, the out-of-bag error rate is 28.57%. All 14 patients in the training set were accurately classified with the predictive model trained by random forest; in the testing set, the AUC is 1.0, the accuracy rate is 100%, the sensitivity is 100%, and the specificity is 100% (Fig. 5b). However, the predictive model with best performances should be further validated in large cohort study. The miRNAs included in each model were listed in supplementary Table 3.

### Validation of miRNAs in patients with different reproductive outcomes

**Fig. 6:**
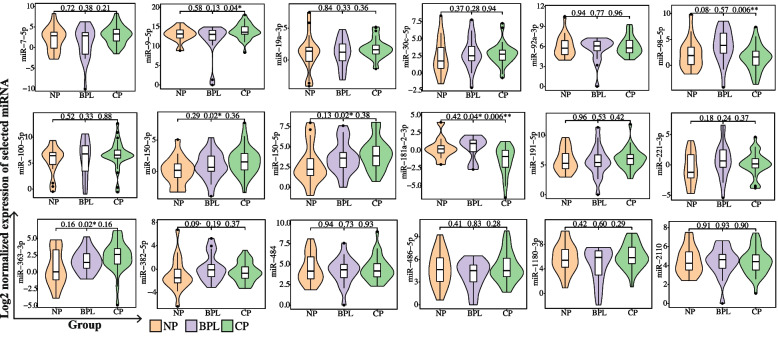
RT-qPCR validation of the selected miRNAs. Relative expression of miRNAs between the patients with different outcomes on the ET day. The mean Ct values of each sample can be found in Supplementary Table 4

A total of 158 patients are recruited during the study period. 18 patients were diagnosed with BPL, 40 with NP, 99 with CP, and 1 with ectopic pregnancy. We matched the BPL, NP, and CP group patients at a ratio of 1:1:2 to control the confounding factors by the PSM model. A total of 72 patients (18 BPL patients, 18 NP patients, and 36 CP patients) were matched and included in the miRNA RT-qPCR validation. Finally, a total of 68 patients were included in the quantitative analysis after excluding 4 early miscarriage patients in further follow-up. The clinical characteristics of the three groups are summarized in Table 1 (right four columns). Age, endometrial thickness, BMI, number of transferred embryos, and embryo stage are not significantly different between the three groups. A total of 18 miRNAs (miR-100-5p, miR-1180-3p, miR-150-3p, miR-150-5p, miR-181a-2-3p, miR-191-5p, miR-19a-3p, miR-2110, miR-221-3p, miR-30c-5p, miR-363-3p, miR-382-5p, miR-484, miR-486-5p, miR-7-5p, miR-92a-3p, miR-9-5p, miR-98-5p) are validated by RT-qPCR. Results showed that the expression of plasma miR-181a-2-3p is significantly increased in patients with BPL (*P*=0.006) and NP (*P*=0.04) compared to CP; the expression of plasma miR-9-5p is significantly decreased in BPL compared to CP (*P*=0.04); the expression of plasma miR-150-3p is significantly decreased in NP compared to CP (*P*=0.02); the expression of plasma miR-150-5p is significantly decreased in NP compared to CP (*P*=0.02); the expression of plasma miR-98-5p is significantly increased in BPL compared to CP (*P*=0.006), the expression of plasma miR-98-5p is increased in BPL compared to NP though without a statistical difference (*P*=0.08); the expression of plasma miR-363-3p is significantly decreased in NP compared to CP (*P*=0.02); the expression of plasma miR-382-5p was increased in BPL compare to NP without a significant statistical difference (*P*=0.09); the other miRNAs are not significantly differentially expressed between the three groups (all *P* values > 0.1). The expression of the validated miRNAs is shown in Fig. 6. The mean Ct values of each sample can be found in Supplementary Table 4.

## Discussion

This study analyzed the dynamic plasma miRNA profiles during the peri-implantation period in patients with different reproductive outcomes, established a predictive model for BPL by machine learning methods, and validated the sequencing results by RT-qPCR. Results showed plasma miRNAs profiles are dynamically changed during the peri-implantation period and are differentially expressed in patients with different reproductive outcomes. Plasma miRNA profiles at ET day are promising in predicting BPL.

Recent studies indicate that embryos' fate following embryo transfer is possible to be predicted before ET. Here, we drew a figure of the embryo fate timeline that describes the embryos' fate continuously following ET (Supplementary Figure 1). As the figure shows, the embryo results in NP (negative pregnancy) have not implanted; the embryo results in BPL starting the implantation process while have not finalized its implantation to the visible gestational sac stage; the embryo results in EM (early miscarriage) losing its pregnancy when a gestational sac is visualized but before 12 gestational weeks (GW); embryo results in late miscarriage lost its pregnancy after 12 GW. Most previous studies focused on implantation failure and clinical miscarriage, either including BPL with negative pregnancy as implantation failure or including BPL with early miscarriage as early pregnancy loss; BPL is seldom studied separately. Unique biochemical characteristics of BPL remain to be investigated.

Circulating miRNAs are associated with pregnancy complications such as recurrent pregnancy loss [34], gestational diabetes [35], preeclampsia [36, 37], small-for-gestational-age births [38]. MicroRNAs are highly desirable as non-invasive biomarkers to predict implantation [39]. However, circulating miRNA indicators for BPL have not been studied. Our miRNA-seq data analyzed the differential dynamic miRNA profiles in patients with different reproductive outcomes during the peri-implantation period. The results highlighted the non-invasive value of plasma miRNAs in predicting BPL. RT-qPCR further validated that plasma miR-150-3p, miR-150-5p, miR-98-5p, miR-363-3p miR-9-5p, and miR-181a-2-3p is differentially expressed between patients with different reproductive outcomes. MiR-150-5p is reported to mediate extravillous trophoblasts migration and angiogenesis [40], miR-150-5p and miR-150-3p is expressed abnormally in failed pregnancy [20], recurrent implantation failure (RIF) [41], and endometriosis [42]. MiR-363 is reported to regulate angiogenesis during pregnancy, and its expression is associated with preeclampsia [43] and RIF [44]. While miR-363-3p is reported to be a hemolysis-susceptible miRNA [45], we suggest caution in the interpretation of miR-363-3p as a reproductive biomarker. MiR-98 is involved in rat embryo implantation during the peri-implantation period [46]; miR-98 in bovine intrauterine extracellular vesicle (EV) regulates endometrial immune responses for implanting conceptuses [47]. Abnormal expression of human decidua miR-98 is associated with miscarriage [48]. Our study validated that the expression of miR-9-5p is significantly downregulated in BPL patients compared to CP patients. A previous study reported that plasma miR-9-5p was downregulated from 6 GW to 23 GW [21]. Our study found that miR-9-5p is time-dependent during the peri-implantation period, with a decreasing tendency in CP patients. Though miR-9-5p is not reported to be associated with human embryo implantation, miR-9-5p promotes the proliferation and migration of endometrial stromal cells in endometriosis [49], miR-9 also plays a role in creating a receptive microenvironment during implantation in pigs [50]. Our study validated that miR-181a-2-3p is elevated in NP and BPL patients compared to CP patients and significantly increases during the peri-implantation period in CP patients. However, no studies have reported the role of miR-181a-2-3p in regulating embryo implantation. Though miR-382-5p, miR-100-5p, miR-1180-3p, miR-191-5p, miR-19a-3p, miR-2110, miR-221-3p, miR-30c-5p, miR-484, miR-486-5p, miR-7-5p, and miR-92a-3p are not validated to be significantly differentially expressed between the three groups. Previous studies found some of the miRNAs are important for embryo implantation, such as the endometrium-derived EV miR-100-5p, which promotes trophoblast functions during embryo implantation [51]. The plasma miR-100-5p is significantly elevated in patients with recurrent miscarriages and decreased in patients with failed pregnancies compared to clinical pregnancy [52]. However, the qPCR experiments of our study did not detect significant differential expression of plasma miR-100-5p between patients with different reproductive outcomes; the different results might be due to individual differences, sample size, and statistical methods. MiR-191-5p is associated with embryo quality [53] and is upregulated in the culture media of implanted human embryos [54]. MiR-19a-3p mediates the regulation of trophoblasts' migration and proliferation [55]. MiR-221-3p is upregulated in the plasma of patients with recurrent pregnancy loss [34], and it regulates trophoblast growth, migration, and invasion [56]. MiR-30c is a marker of blastocyst implantation potential [17, 57]; miR-30c-5p in uterine fluid during the implantation phase is significantly downregulated in RIF patients [44]. The plasma miR-486-5p at ET day is significantly decreased in patients with recurrent miscarriage, and its expression level is potentially predictive of clinical pregnancy [52]. A recent study reported that miR-486-5p in uterine fluid during implantation was significantly downregulated in RIF patients [44]. MiR-7 plays a role in trophoblasts' invasion via the TGF-β-Smad pathway [58]. MiR-92a-3p plays a role in endometriosis [59] and is associated with endometrial receptivity [60] and trophoblast invasion [61].

The plasma miRNA profiles are under dynamic changes from early pregnancy to late pregnancy [21]. However, no study reports the dynamic changes of plasma miRNA in the peri-implantation period. Our time course analysis found that 15 miRNAs were differentially expressed across the analyzed time-points. Overall, the dynamic change of plasma miRNAs and the different time-dependent tendency between the BPL and CP patients suggests an embryo-maternal communication during the peri-implantation period, which might account for biochemical pregnancy loss.

The WGCNA analysis identified 13 miRNA co-expression modules. Some modules are relevant to reproductive outcome (purple, magenta, blue, and green modules), female age (pink, green, and turquoise modules), embryo factor (pink, blue, and green modules), BMI (purple module), and hCG levels (green module). Age, embryo factor, BMI, and hCG levels are closely related to reproductive outcomes. The close module-module relationships indicate that the plasma miRNA expression profile may reflect the comprehensive situation affecting reproductive outcomes.

We explored the differential dynamic miRNA profiles during the peri-implantation period in patients with different reproductive outcomes and identified BPL-indicative miRNAs. We found the non-invasive value of plasma miRNAs in predicting BPL. Nevertheless, the following limitations should be underlined: First, the sample size used for predictive model construction in the miR-seq stage is relatively small; Second, we did not investigate the underlying molecular mechanisms that might explain the role of the significant miRNAs in embryo implantation; Third, only the plasma miRs were sequenced, however, the miRNAs profile in buffy coat samples, the intrauterine fluid samples, or the endometrial samples, were not sequenced, therefore, it is impossible to determine the main source of plasma indicator miRNAs for BPL prediction; Fourth, we did not measure the hemolysis of each sample to exclude the effect of hemolysis on the expression of circulating miRNA. An additional prospective study with larger sample size and mechanism research is required before the miRNA biomarkers can be confidently used in clinical settings. Moreover, future prospective clinical studies that use a quick miRNA qPCR panel to detect the BPL-indicative plasma miRNAs at one time may provide an efficient non-invasive and early diagnostic tool at the ET cycle.

## Conclusions

In conclusion, our findings identify altered dynamic miRNA profiles in BPL patients and suggest a possible role of plasma miRNAs as novel and non-invasive biomarkers for BPL. It would be helpful to predict the reproductive outcomes in the ET cycle and is essential for providing the pathological basis, prevention, and treatment of BPL.

### Supplementary Information

**Supplementary file 1.**

**Supplementary file 2.** 

**Supplementary file 3.**

**Supplementary file 4.**

**Supplementary file 5.**

## Data Availability

The datasets supporting the conclusions of this article are available in the supplementary materials and the miRNA-seq data were deposited in the NCBI Gene Expression Omnibus (GSE211748).

## Abbreviations

NP: Negative pregnancy
BPL: Biochemical pregnancy loss
EM: Early miscarriage
CP: Clinical pregnancy
IVF: In vitro fertilization
ET: Embryo transfer
qRT-PCR: Reverse transcription-polymerase chain reaction
ART: Assisted reproductive technology
hCG: Human chorionic gonadotropin
PGD: Preimplantation genetic diagnosis
WGCNA: Weighed gene co-expression network analysis
PCA: Principal Components analysis
GO: Gene ontology
FC: Fold change
