# Supplementary figures and images for: Specific plasma microRNA profiles could be potential non-invasive biomarkers for biochemical pregnancy loss following embryo transfer
Source: BMC Pregnancy Childbirth. 2024 May 8;24:351. doi: 10.1186/s12884-024-06488-x (PMC11080217; doi:10.1186/s12884-024-06488-x)

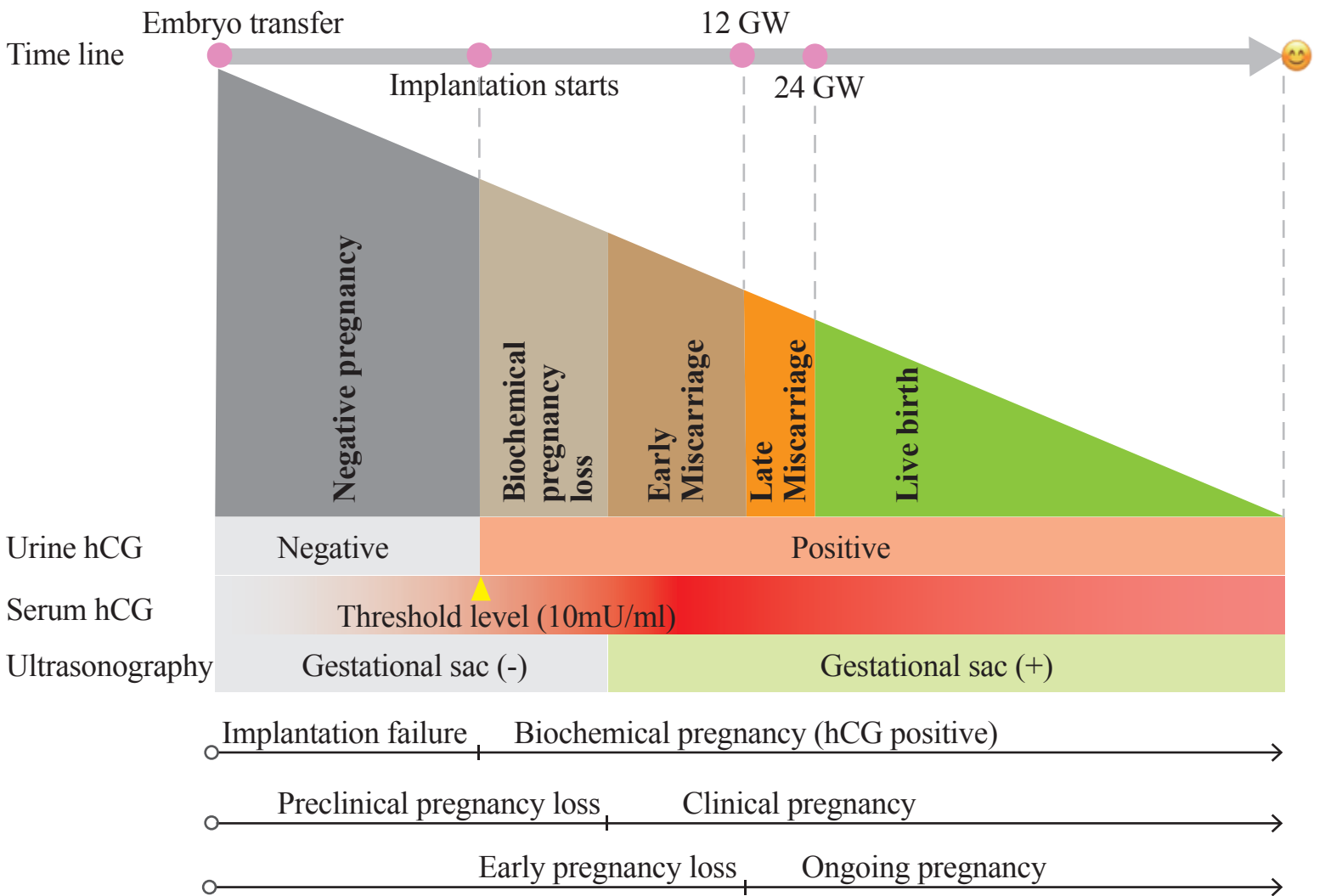

Supplement: Supplementary file 1 [file 12884_2024_6488_MOESM1_ESM.pdf]
